# Powerful Identification of *Cis*-regulatory SNPs in Human Primary Monocytes Using Allele-Specific Gene Expression

**DOI:** 10.1371/journal.pone.0052260

**Published:** 2012-12-26

**Authors:** Jonas Carlsson Almlöf, Per Lundmark, Anders Lundmark, Bing Ge, Seraya Maouche, Harald H. H. Göring, Ulrika Liljedahl, Camilla Enström, Jessy Brocheton, Carole Proust, Tiphaine Godefroy, Jennifer G. Sambrook, Jennifer Jolley, Abigail Crisp-Hihn, Nicola Foad, Heather Lloyd-Jones, Jonathan Stephens, Rhian Gwilliam, Catherine M. Rice, Christian Hengstenberg, Nilesh J. Samani, Jeanette Erdmann, Heribert Schunkert, Tomi Pastinen, Panos Deloukas, Alison H. Goodall, Willem H. Ouwehand, François Cambien, Ann-Christine Syvänen

**Affiliations:** 1 Department of Medical Sciences, Molecular Medicine and Science for Life Laboratory, Uppsala University, Uppsala, Sweden; 2 Department of Human Genetics, McGill University, Montréal, Canada; 3 INSERM UMRS 937, Pierre and Marie Curie University and Medical School, Paris, France; 4 Department of Genetics, Texas Biomedical Research Institute, San Antonio, Texas, United States of America; 5 Department of Haematology, University of Cambridge, Cambridge, United Kingdom; 6 National Health Service Blood and Transplant, Cambridge Centre, Long Road, Cambridge, United Kingdom; 7 Wellcome Trust Sanger Institute, Wellcome Trust Genome Campus, Hinxton, Cambridge, United Kingdom; 8 Klinik und Poliklinik für Innere Medizin II, Universität Regensburg, Regensburg, Germany; 9 Department of Cardiovascular Science, University of Leicester, Leicester, United Kingdom; 10 Leicester NIHR Biomedical Research Unit in Cardiovascular Disease, Glenfield Hospital, Leicester, United Kingdom; 11 Medizinische Klinik II, Universität zu Lübeck, Lübeck, Germany; Karolinska Institutet, Sweden

## Abstract

A large number of genome-wide association studies have been performed during the past five years to identify associations between SNPs and human complex diseases and traits. The assignment of a functional role for the identified disease-associated SNP is not straight-forward. Genome-wide expression quantitative trait locus (eQTL) analysis is frequently used as the initial step to define a function while allele-specific gene expression (ASE) analysis has not yet gained a wide-spread use in disease mapping studies. We compared the power to identify *cis*-acting regulatory SNPs (*cis*-rSNPs) by genome-wide allele-specific gene expression (ASE) analysis with that of traditional expression quantitative trait locus (eQTL) mapping. Our study included 395 healthy blood donors for whom global gene expression profiles in circulating monocytes were determined by Illumina BeadArrays. ASE was assessed in a subset of these monocytes from 188 donors by quantitative genotyping of mRNA using a genome-wide panel of SNP markers. The performance of the two methods for detecting *cis*-rSNPs was evaluated by comparing associations between SNP genotypes and gene expression levels in sample sets of varying size. We found that up to 8-fold more samples are required for eQTL mapping to reach the same statistical power as that obtained by ASE analysis for the same rSNPs. The performance of ASE is insensitive to SNPs with low minor allele frequencies and detects a larger number of significantly associated rSNPs using the same sample size as eQTL mapping. An unequivocal conclusion from our comparison is that ASE analysis is more sensitive for detecting *cis*-rSNPs than standard eQTL mapping. Our study shows the potential of ASE mapping in tissue samples and primary cells which are difficult to obtain in large numbers.

## Introduction

Owing to the rapid advances in genotyping technology a large number of genome-wide association studies have been performed during the past five years to identify associations between SNPs and human complex diseases and traits [1]. These studies have detected genome-wide significant association signals for about 2200 loci with at least 240 diseases or traits [2], [3]. As the majority of the associated SNPs are located outside protein-coding genes, the assignment of a functional role for the identified disease-associated SNP is not straight-forward. Genome-wide expression quantitative trait locus (eQTL) analysis of tissues or cell types that are relevant for a disease or trait of interest is frequently used as the initial step to define a function for disease-associated SNP alleles [4], [5], [6]. An association between a SNP and the expression level of a gene indicates that this SNP, or a variant in strong linkage disequilibrium (LD), may be involved in the regulation of the expression of the gene. Genome-wide eQTL analysis will identify *cis*-acting rSNPs located on the same chromosome nearby the target gene and *trans*-acting rSNPs that are usually located on a different chromosome or distant from the target gene. The *cis*-regulatory SNPs normally have stronger effects and are therefore easier to identify by eQTL mapping than *trans*-acting SNPs. Consequently, c*is*-regulatory SNPs identified in a relevant tissue are functional candidates for further investigation of disease-causing genetic variants.

Although quantitative, genome-wide allele-specific gene expression (ASE) analysis has previously been introduced as an alternative to eQTL mapping of *cis*-regulatory SNPs associated with human diseases and traits [7], [8], it has not yet gained a wide-spread use in disease mapping studies. One reason might be that the power of ASE has not been systematically demonstrated in comparison with that of the widely used approach of eQTL mapping using gene expression data from hybridization microarrays. The power and precision of ASE analysis stems from the fact that allelic variations in gene expression levels are determined within each sample, instead of variations in total gene expression levels between samples of different genotype as in eQTL analysis. In ASE analysis, using a genome-wide panel of SNP markers, heterozygous SNPs are genotyped both at the RNA and genomic DNA levels, the later being used as reference for the quantification of allelic expression. The aim of the study presented here is to compare the statistical power of identifying *cis*-regulatory SNPs by ASE analysis with that of traditional eQTL mapping. As our study focuses exclusively on the analysis of *cis*-rSNPs, we refer here to eQTL mapping of *cis*-rSNPs as genotypic expression (GTE) mapping. We performed an experimental comparison of ASE and GTE by analyzing gene expression in primary monocytes purified from human peripheral blood samples. Monocytes were analyzed because they are a relevant cellular model for many complex diseases.

By computing Bonferroni-corrected p-values, false discovery rate p-values and by determining the overlap between the SNPs with the strongest association signals identified by both methods in addition to an analysis of the effect of minor allele frequencies upon the power of the methods, we show unequivocally that ASE analysis is more powerful than GTE mapping for identifying *cis*-regulatory SNPs.

## Results

In this study we compared two approaches for genome-wide association analysis of SNPs against gene expression levels. Using GTE mapping, the SNP genotypes are tested for an association with the total gene expression levels, and using ASE analysis, the relative expression levels between the two alleles of a transcript are used as the quantitative phenotype against which the SNP genotypes are analyzed. The results presented here are based on GTE mapping of RNA extracted from 395 monocyte samples and ASE analysis of a subset of 188 monocyte samples from the same donors. The donors are healthy adult blood donors of European origin recruited from the United Kingdom National Blood Service Centre in Cambridge, UK as part of the Cardiogenics Transcriptomic Study. The power of GTE mapping and ASE analysis to detect association signals from SNPs that affect gene expression in the monocytes was explored by comparing the number of SNPs with significant association signals obtained using different p-value thresholds in sample sets of different sizes. To enable comparison of the statistical power between the two approaches the ASE dataset was down-sampled to 95 and 50 samples, and the GTE dataset was down-sampled to 188, 95 and 50 samples. The principles of the two methods are illustrated in Supplementary Figure S1.

### Association analysis to detect *cis*-acting regulatory SNPs

The comparative set of 517K SNP markers for which genotype data was available for all samples were used for association analysis against the total gene expression levels and the ASE levels. After filtering, 12145 probes representing 10059 Refseq transcripts remained for the GTE analysis and 13146 Refseq transcripts remained for the ASE analysis. Thus 544531 and 638845 association tests were performed for GTE mapping and ASE mapping, respectively. By filtering out association tests that were based on too few data values, for ASE we retain 620 k to 438 k tests depending on the sample size and for GTE 428 k to 343 k tests depending on the sample size, see further below. For significance according to Bonferroni at p_corr_ = 0.05 these numbers imply that an observed p-value of 7.8e-8 is required for the complete ASE analysis and for p_corr_ = 0.01 a p-value of 1.6e-8 is required. Similarly, for the complete GTE analysis, an observed p-value of 1.2e-7 for p_corr_ = 0.05 and 2.3e-8 for p_corr_ = 0.01 are required. To avoid inflated false positive rates, both Bonferroni and FDR adjustments require that random sampling or permutation of the data should yield uniformly distributed p-values under the null hypothesis. To verify that the p-values were uniformly distributed, the GTE and ASE data was permuted 500 times by shuffling the sample identifiers once for each iteration. In this way, the connection between genotypes and ASE values are broken while the structure of the data is retained so that phasing and the calculation of the median ASE levels is not affected. According to the permutation tests, the number of p-values in the very low range was inflated in both the GTE and the ASE data. However, filtering out association tests that were based on linear regression analysis of less than four ASE values per genotype group in the ASE analysis and less than three expression values per genotype group in the GTE analysis, abolished the inflation, see QQ-plots in Figure S2.

Table 1 shows the number of significant association signals obtained by GTE mapping and ASE analysis at different p-value thresholds. The association signals are not filtered by linkage disequilibrium for either of the methods. For all sample sizes the ASE method yielded a larger number of significant SNP-transcript associations than the GTE analysis (top panel of Table 1). As the same SNP could be associated with several transcripts, we also examined the number of observed SNP-transcript associations when only the strongest association for each SNP was considered. As before, the ASE method identified a larger number of these SNPs than the GTE analysis at all sample sizes (middle panel of Table 1), and also a larger number of transcripts to which at least one SNP was significantly associated (bottom panel of Table 1). The difference in number of putative *cis*-rSNPs detected with significant association signals between the ASE and GTE methods is most pronounced in the smaller sample sets, where up to 31 times more *cis*-rSNPs and up to nine times more transcripts were detected by ASE analysis using the same sample size and a 5% FDR threshold. As can be seen in Table 1 the advantage of the ASE approach is less pronounced at the larger sample sizes. A larger number of significant associations were also detected by ASE than by GTE when using the stricter Bonferroni cut-off, where only the strongest association signals are detected.

**Table 1:** Comparison of the power to detect *cis*-acting regulatory SNPs using allele-specific expression (ASE) and genotype expression (GTE) analysis.

Type of p-value threshold	Method	Number of samples^a^			
		395	188	95	50
**Total number of significant SNP-transcript associations**					
FDR 5%	ASE	NA	203893	127990	65258
	GTE	31963	16757	6879	2077
	ASE/GTE	NA	12.2	18.6	31.4
FDR 1%	ASE	NA	155205	87523	38685
	GTE	22651	11101	4242	1151
	ASE/GTE	NA	14.0	20.6	33.6
Bonferroni correction p_corr_ = 0.05	ASE	NA	58078	23213	6781
	GTE	9424	4277	1439	390
	ASE/GTE	NA	13.6	16.1	17.4
Bonferroni correction p_corr_ = 0.01	ASE	NA	51383	19357	5191
	GTE	8337	3677	1189	300
	ASE/GTE	NA	14.0	16.3	17.3
**Total number of significantly associated SNPs**					
FDR 5%	ASE	NA	111978	76161	42100
	GTE	24379	13479	5887	1863
	ASE/GTE	NA	8.3	12.9	22.6
FDR 1%	ASE	NA	88837	54758	26284
	GTE	17975	9281	3739	1064
	ASE/GTE	NA	9.6	14.6	24.7
Bonferroni correction p_corr_ = 0.05	ASE	NA	37995	16467	5037
	GTE	8148	3855	1349	370
	ASE/GTE	NA	9.9	12.2	13.6
Bonferroni correction p_corr_ = 0.01	ASE	NA	34131	13876	3881
	GTE	7266	3335	1119	284
	ASE/GTE	NA	10.2	12.4	13.7
**Total number of transcripts with significant SNP associations**					
FDR 5%	ASE	NA	12389	7111	6447
	GTE	5051	3364	1850	740
	ASE/GTE	NA	3.7	3.8	8.7
FDR 1%	ASE	NA	11914	6788	5472
	GTE	3620	2256	1158	423
	ASE/GTE	NA	5.3	5.9	12.9
Bonferroni correction p_corr_ = 0.05	ASE	NA	8723	4395	1984
	GTE	1867	1064	476	165
	ASE/GTE	NA	8.2	9.2	12.0
Bonferroni correction p_corr_ = 0.01	ASE	NA	8223	3994	1607
	GTE	1712	948	407	133
	ASE/GTE	NA	8.7	9.8	12.1

a Median values of 10 runs for the random sample subsets. The top panel show the number of significant SNP-transcript associations, the middle panel show the number of significantly associated SNPs when counting only the best SNP-transcript association for each SNP, and the bottom panel show the number of transcripts that have at least one significantly associated SNP. Both the Bonferroni and FDR p-value thresholds are used. ASE/GTE = Ratio between number of significant ASE and GTE associations.

The number of significantly associated rSNPs based on the FDR p-values indicates that up to eight times fewer samples are required for ASE analysis to achieve the same statistical power as GTE mapping, at least for the sample sizes analyzed in this study. From the bottom panel of Table 1 it can also be seen that in the ASE analysis almost all transcripts show significant ASE when using the 5% FDR threshold, and that over half of them have significant ASE when using the stricter Bonferroni p_corr_ = 0.05 cut-off. Most of the 13146 Refseq transcripts that were analyzed for ASE association show some degree of allele-specific expression, but it was unexpected that the ASE method would detect significant associations with transcripts showing only marginal effect sizes. However, formal statistical significance does not necessary imply that the effect is large enough to be biologically relevant. In a biological application one needs to consider having a cutoff for the minimal ASE-level as well as the p-value.

### Location of *cis*-regulatory SNPs in gene regions

The fraction of significantly associated rSNPs compared to all SNPs is plotted in Figure S3 according to their relative distance to the transcription start and termination sites. In both the ASE and GTE data there is a peak in the abundance of putative rSNPs close to the transcription start site and immediately after the transcription termination site. There is also an over-representation of rSNPs in the regions before the start site and after the termination site compared to the transcribed regions. The enrichment is more evident at the lower p-value thresholds obtained by the Bonferroni correction, indicating that the strongest effect occurs by SNPs located closest to the transcription start and termination sites.

### Effect of increased transcript flanking regions and less stringent filtering

When including SNPs within 500 kb regions flanking the transcripts instead of 100 kb for GTE mapping, to facilitate identification of SNPs located more distantly from the transcripts, the number of significant association signals decreased by one third using 5% FDR and all the samples. However, the number of significantly associated transcripts is similar with 805 associated transcripts lost in the 100 kb region and 913 new associated transcripts found in the extended region. When the same expanded 500 kb flanking regions were included in the ASE analysis a similar amount of significant association signals as for 100 kb flanking regions were observed, although the p-values were corrected for almost four times as many tests. Here, 127 transcripts associations were lost in the 100 kb region and 169 new transcripts were found in the extended region. For both methods it is worth investigating different sizes of flanking regions to define the optimal balance between the number of association tests and the number of false negative rSNPs. Additionally, the ASE levels were determined using the less stringent requirement of three informative SNPs instead of five for calculating the ASE level which yielded 1343 additional transcripts for analysis. In this case a small decrease in number of significant association signals was observed due to slightly larger number of tests and a lower signal to noise ratio.

### Overlap in SNPs detected by ASE and GTE analysis

**Figure 1:**
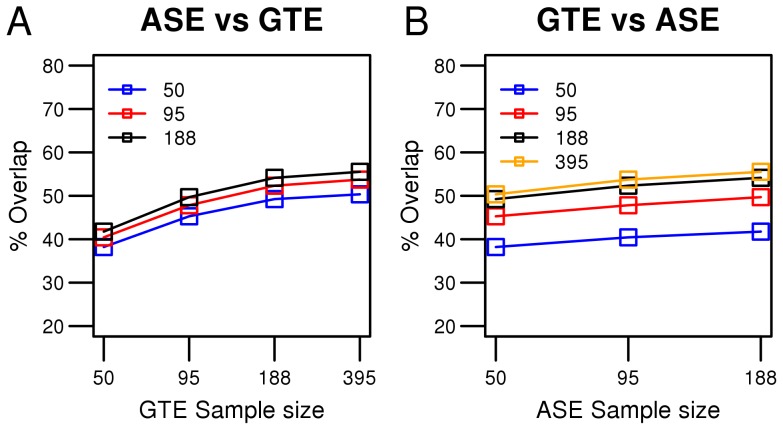
Overlap of significantly associated rSNPs identified by ASE and GTE. The percentage of overlapping rSNPs detected by allele-specific expression (ASE) and genotype expression (GTE) analysis is plotted for varying numbers of samples. The top 9536 SNPs from the GTE analysis are compared with the top 38203 SNPs from the ASE analysis, which corresponds to a Bonferroni threshold of p = 0.05 for a GTE sample size of 395 and an ASE sample size of 188. The p-value cut-offs were adapted so that the same SNP top-list sizes were obtained at all sample sizes for both GTE (p-value of 1.17E-7, 1.06E-4, 1.93E-3, 6.12E-3 for n = 395, n = 188, n = 95, and n = 50 respectively) and ASE (p-value of 8.06E-8, 9.35E-5, 4.90E-3 for n = 188, n = 95, and n = 50 respectively). The vertical axes show the percentage of SNPs in the top-lists detected by both GTE and ASE analysis and the horizontal axes show the number of samples analyzed using GTE and ASE, respectively. The percentage overlap is calculated by dividing the number of overlaps with the number of top SNPs in the GTE analysis. In (A), each line shows the effect on the number of overlapping SNPs detected by ASE analysis of a specific sample size when the sample size in GTE analysis was increased. In (B), each line shows the effect on the number of overlapping rSNPs detected by GTE analysis of a specific sample size when the samples size in ASE analysis is increased.

To further compare the power of GTE and ASE mapping, we determined the overlap in *cis*-rSNPs detected by the two methods at different sample sizes. The overlap in transcripts targeted by the GTE arrays and the requirement of heterozygous SNPs in ASE transcript windows sets an upper limit to the number of overlapping rSNPs that are detectable by both methods. In our data between 150 k and 200 k SNPs were eligible for assessing overlapping results between the two methods, depending on which sample sizes were compared. The SNPs were sorted according to p-value and the top 9536 SNPs from the GTE analysis were compared with the top 38203 SNPs from the ASE analysis, corresponding to a Bonferroni p_corr_ = 0.05 threshold for GTE sample size 395 and ASE sample size 188. The overlap was calculated for both methods and all sample sizes, see Figure 1. The p-value cut-offs were adapted so that the same SNP top-list sizes were obtained at all sample sizes for both GTE and ASE.

Using this approach it can be assessed if an increase in sample size would yield more overlapping rSNPs or if most of the rSNPs have already been identified. In Figure 1A the rSNP overlap increases only marginally as the ASE sample size increases, while a relatively large gain of additional rSNPs is obtained by increasing the GTE sample size. Figure 1B is another representation of the same data showing the comparison in the opposite way. Figure 1 show that the gain in overlapping low p-value SNPs is larger when increasing the GTE sample size than when increasing the ASE sample size. This observation indicates that ASE is a more powerful technique as it has found most of the overlapping SNP already at low sample sizes. Moreover, Figure 1 show that high-ranking rSNPs detected by one of the two methods are to a high degree also detected at high rank by the other method and that the two methods are in good agreement with each other for low p-value rSNPs.

### Effect of minor allele frequency of SNPs

**Figure 2:**
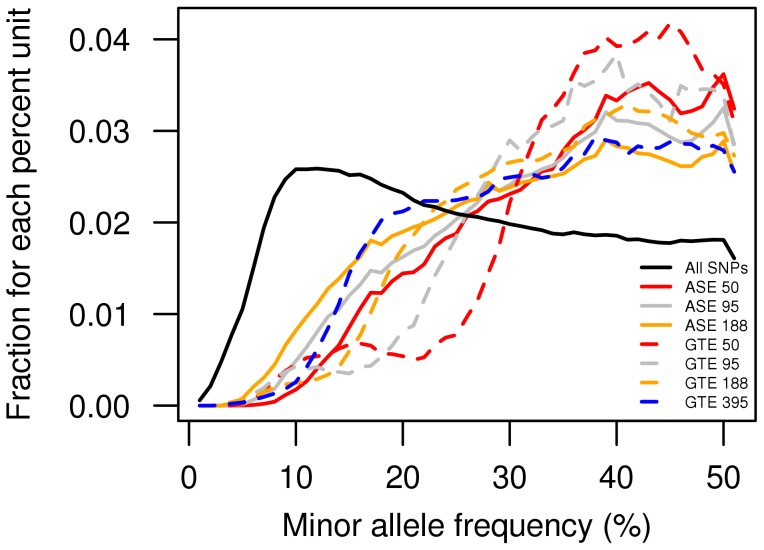
The ability of ASE and GTE analysis to detect significantly associated rSNPs at different MAF. Fractions of rSNPs are shown for different minor allele frequencies (MAF) with significant association signals according to a Bonferroni-corrected p-value of 0.05. Each data point underlying the curves represents the fraction of significant associations within a 1% MAF bin. Sliding 5% MAF window averages are plotted for different sample sizes analyzed by ASE and GTE. Both methods detect a lower fraction of low frequency rSNPs, compared to the fraction of all the SNPs at the same frequency (black line). The ASE method detects a higher fraction of the SNPs (solid lines) with a MAF <15% than GTE (dashed lines) regardless of sample size except for the largest GTE sample set.

Finally, to examine how the minor allele frequency (MAF) of the SNPs affects the power of the two methods to detect putative *cis*-rSNPs, the genotype data was divided into bins, with all SNPs within a MAF window of 1% unit between 0% and 50% in each bin. The fraction of significantly associated rSNPs within each 1% MAF window was determined based on Bonferroni-corrected p-values at a p_corr_ = 0.05 threshold. Figure 2 shows the fraction of rSNPs detected by GTE and ASE at different sample sizes plotted as a function of the MAF. As can be seen in Figure 2, there is a correlation between the allele frequency of the SNPs and the detected fraction of rSNPs, with a lower fraction of detected rSNPs with lower MAF, as would be expected. However, the sliding widows of average MAF-values also show that the ASE analysis (solid lines) detects a larger fraction of rSNPs with MAF <15% than GTE mapping (dashed lines) regardless of sample size, with the exception of the largest GTE sample size. When absolute numbers are compared the difference is much more profound.

## Discussion

In our study we show by three approaches, *i.e.* using adjusted p-value thresholds obtained by the Bonferroni and FDR approaches, by analysis of top-list overlaps and MAF analysis to assess the relative power of ASE analysis and eQTL mapping of *cis*-rSNPs (GTE mapping), that ASE analysis is more powerful than GTE analysis for mapping *cis*-regulatory SNPs. The high power of ASE originates from the fact that using ASE, the relative expression levels of the two alleles of a transcript are measured in the same sample, and thus *trans*-acting regulatory factors and other differences in gene expression between samples due to exposure of the samples to different environmental conditions are controlled for. A limitation of mapping rSNPs using ASE, compared to traditional GTE mapping is that ASE would not be applicable to the rare cases of transcripts lacking heterozygous SNPs and also that transcripts without enough heterozygous SNP coverage on the genotyping chip would be excluded. This limitation can be partly circumvented by using the sum of the fluorescence signals from the two alleles of each SNP as a quantitative measure for total gene expression levels. Also, our ASE approach is only sensitive for variants altering transcription and not those affecting splicing, while it is possible to find these using eQTL approaches if the microarray used have large enough resolution.

To perform a comparison that was as fair as possible, we analyzed exactly the same SNPs using both methods. For these SNPs linkage disequilibrium (LD) was not considered as it is not unequivocal to distinguish between SNPs that are in LD and SNPs that are not. For the purpose of comparing the relative power of ASE and GTE, a comparison without consideration of LD is adequate. The number of transcripts analyzed in the comparison was also similar, although not identical. The 15% higher number of transcripts that were eligible for ASE analysis has no impact on the conclusion from our study, but instead reflects another advantage of ASE, which is that the ASE approach does not require annotated transcripts and probe design prior to performing an experiment. The flexibility of avoiding predesigned probes and the high power of ASE using SNP genotyping is shared with second generation transcriptome sequencing (RNA-seq), where both total gene expression and allele-specific expression levels can be determined in the same data set. Thus ASE will be an informative approach for RNA-seq data, particularly for samples with available genotype data. An additional advantage of RNA-seq compared to ASE analysis using SNP genotyping is that alternatively spliced transcripts and strand specific gene expression can be detected, provided that the sequencing coverage is sufficient.

In a previous study we have shown the low level of noise in ASE data for 8000 genes using a closely related genotyping assay [9]. In that study, the median correlation for allelic fractions in DNA (Allele1/(Allele1+Allele2)) between replicas was 0.9969 (range 0.9934–0.9986) and for RNA 0.9956 (range 0.9779–0.9984) in 197 samples. In our data the SD for gDNA allele ratios (A1/(A1+A2)) over a region is very small, with an average of 0.014 (range 0.002–0.071). For RNA the SD over a region can only be calculated per sample as the allelic ratio in RNA is expected to vary depending on differences between samples in allele specific expression levels. The SD for the allelic ratio over a region per sample is therefore higher (average 0.10, range 0.00–0.31). Additionally, we have determined the standard deviations for the ASE-levels between samples over the regions analyzed in the current study. For 12% of the transcript regions over half of the samples have an ASE-level with higher standard deviations than the mean. These could be regions with either a considerable level of alternative splicing, indications of phasing errors or a high error rate in the genotyping fluorescence signal. Of these regions, 9% persist even when considering only regions with an associated p-value below the Bonferroni threshold. If there would be phasing errors, the signals could be false positives, but the association signal would be strengthened in most cases when the phasing is corrected. A similar reasoning is true for splice variants, in which a smaller region from which the splice has been excluded site would probably strengthen the association signal. Functional assessment of disease-associated variants by ASE analysis has been applied to lymphoblastoid cell lines [10], osteoblasts [10], fibroblasts [11], and T-cells [12]. By ASE analysis of a large set of primary human monocytes, we show here that as little as 50 samples may be sufficient for robust mapping of many *cis*-regulatory genetic variants. The high power of ASE is reassuring for functional refinement of SNP identified by genome-wide association studies of disease and traits in relevant tissue or cell samples, which in many cases are difficult to obtain in large numbers.

## Materials and Methods

### Ethics Statement

The study is approved by the Cambridgeshire 1 Research Ethics Committee.

### RNA and DNA samples

Circulating monocytes were collected from healthy adult blood donors of European origin recruited from the United Kingdom National Blood Service Centre in Cambridge, UK as part of the Cardiogenics Transcriptomic Study. Samples from patients with a recent or acute illness were excluded. DNA was extracted from peripheral blood leukocytes using the guanidine hydrochloride - chloroform method followed by quantification by PicoGreen (Invitrogen, Paisley, UK). CD14+ magnetic microbeads (autoMACS Pro, Miltenyi Biotec, Bergisch Gladbach, Germany) were used to isolate monocytes from whole blood. RNA was extracted from cell pellets of freshly isolated monocytes by homogenization with Trizol-reagent (Invitrogen, Paisley, UK), chloroform-ethanol extraction and purification using Qiagen RNAeasy columns and reagents, followed by on-column DNase treatment. cDNA was synthesized using reagents from the Illumina TotalPrep RNA Amplification Kit. Synthesis of cDNA was performed according to the protocol provided by the supplier, except that the poly-dT primers were substituted by random decamers (Applied Biosystems, Carlsbad, California, US).

### Genotype expression (GTE) analysis

Human-Ref 8 v3.0 arrays (Illumina Inc) containing 24526 probes were used to generate the gene expression data for GTE mapping essentially as described previously in [13]. A total of 31535 transcripts were extracted from Refseq hg18 after removing all non-unique transcripts, including both genes and some lncRNAs. These Refseq transcripts were used to map the expression probes from the Human-Ref v3.0 arrays to their corresponding transcripts. Probes that did not generate expression signals above the detection score threshold, corresponding to a p-value of 0.05 were filtered out, leaving data from 12145 probes for the GTE analysis. Of the corresponding transcripts there was 22% that had overlaps with other transcripts. The raw GTE expression data was transformed using variance stabilization transformation (VST) and subjected to quantile normalization using the R packages Lumi and Beadarray.

Of the monocyte samples, 247 were subjected to genome-wide genotyping for GTE mapping with Human 660 Quad BeadChips (Illumina Inc, San Diego, California, US) and 188 samples were genotyped for the ASE analysis by the Infinium II assay with Human 1.2 M Duo custom BeadChips v1 (Illumina Inc). To apply a comparative set of markers for the comparison of GTE and ASE, only those 517 K markers that were present on both chips were used for GTE and ASE mapping. Genotypes from the 660 Quad BeadChips were called in Illuminus [14] using default parameters, excluding genotypes with a posterior probability <0.9 of being correct.

### Allele-specific expression (ASE) analysis

Genotyping of the 188 RNA samples for ASE analysis was performed using 1.2 M Duo custom BeadChips v1 (Illumina Inc). The cDNA and genomic DNA (gDNA) from each sample were genotyped on the same BeadChip to minimize experimental variation. Genotypes were called in gDNA using Genome Studio version 2009.2 (Illumina Inc.). A call rate of 0.99 was set as the threshold for genotype calls. A quadratic function was employed to normalize the raw two-colour fluorescence signals from the assay with respect to the dependency between signal intensity and the relative signal intensities of the two fluorophores corresponding to the two SNP-alleles. Phasing of the SNP data was performed by Impute2 [10] on the complete SNP data set, including both heterozygote and homozygote genotypes from the 1.2 M BeadChips to determine the haplotypes in gDNA for each individual sample. Using phased samples the ASE levels can be assigned in bidirectionally to indicate which of the two alleles of each SNP is over-expressed according to the highest haplotype probability obtained from the phasing. ASE levels were calculated for each heterozygote SNP as the difference in allele fractions between cDNA and gDNA according to the equation: Allele1_cDNA_/(Allele1_cDNA_+Allele2_cDNA_) – Allele1_gDNA_/(Allele1_gDNA_+Allele2_gDNA_). The same 31535 transcripts as in GTE analysis were extracted from Refseq genes [15] release 47 for hg18. After filtering out transcripts with fewer than five informative SNPs, 13146 Refseq transcripts remained for the ASE analysis, out of which 28% had overlaps with other transcripts. A SNP is informative for detecting ASE if at least one sample is heterozygous for that SNP. The average number of informative SNPs per transcript was 22.9 (median 13). The ASE levels of the phased SNPs where then averaged over the corresponding transcript window to obtain the ASE level for the expressed haplotype in each sample. The ASE levels were then used in the association tests to detect significantly associated rSNPs as detailed in the section Statistical Analyses below.

Because the genotype calling from the 660 Quad and the 1.2 M Duo BeadChips differed slightly, the result from the genotype calls were compared for the overlapping 517 K SNPs in the 188 samples that where genotyped on both chip types. The genotypes in these data sets were found to differ at as little as 0.0056% of the bases and only 0.19% of the SNPs were in any way affected by this minor difference in genotype calls.

The allelic genotyping data and expression in this paper are in the process of being deposited in the EGA with accession number EGAS00000000119 (https://www.ebi.ac.uk/ega).

### Statistical analyses

The set of 517 K genotyped SNPs that were shared between the GTE and ASE analysis was used to identify rSNPs. GTE mapping and ASE analysis was performed using the genotypes of all SNPs located in a region of 100 kb upstream and downstream of the transcript as well as within the region of the transcript itself. For ASE these SNPs were tested by linear regression for associations between SNPs and ASE levels essentially as described by Pastinen et al [7]. In brief, the heterozygous samples are treated as two separate groups depending on by the phasing, and the homozygous samples are included as one group in the linear regression to represent the baseline ASE level. This analysis differs from that for GTE where the heterozygous samples form one group and the homozygous samples form two different groups. For GTE, association of expression levels with genotypes was calculated using linear regression with age and sex of the monocyte donors as confounding factors. The statistical analysis was performed using R. The Bonferroni [16] and the Benjamini-Hochberg [17] false discovery rate adjustments were applied to correct for multiple testing. The Bonferroni correction can be expressed as: p_corr_ = p*n, where p_corr_ is the corrected p-value and n is the number of tests. The FDR adjusted p-values were calculated by a linear step-up adjustment [18] in the following fashion: p_corr_ = (m/i)*p, where m is the number of tests and i is the ranking of p-values among all observed p-values when they are sorted in increasing order. FDR p-values that do not monotonically increase were finally replaced with the smallest observed FDR value for any higher ranked probe.

## Supporting Information

Figure S1 **Schematic picture of the principles of GTE and ASE analysis.** In GTE the total expression level is measured by a specifically designed expression probe usually located in a transcript. The effect of different genotypes (shown in yellow) can then be tested against the total expression level of each sample to find regulatory SNPs using a linear regression association test. In ASE the allele-specific expression level is measured by the difference in fluorophore signal intensity between the two alleles in the same sample. The average level is calculated for all heterozygous SNPs in the region. This value is then used when testing whether there is a significant effect of the tested genotype (shown in yellow) in a similar association test as in GTE.
(TIF)

Figure S2 **QQ-plots for the permuted p-value distribution compared with a uniform distribution.** In Panel A–C, QQ-plots are shown for ASE and in panel D–G QQ-plots are shown for GTE using filtering that removes SNPs with few data points in each genotype group. The permuted data are expected to yield uniformly distributed p-values under the null hypothesis and should therefore follow the red line representing the uniform distribution, which is also the case. However, without filtering, the distribution is starting to skew towards inflated p-values prior to the Bonferroni correction threshold is reached, data not shown.
(TIF)

Figure S3 **Fraction of rSNPs in relation to the position of TSS and TTS.** The fraction of significant association signals compared to the total number of SNPs in 1 kb bins at different distances to the transcription start site (A, B, F, E) and transcription termination site (C, D, G, H) is shown for ASE using 188 samples and for GTE using 395 samples. In A–D the FDR 5% threshold is used and in E–H the Bonferroni 0.05 threshold. All figures show peaks around the start and termination sites.
(TIF)
