# Supplementary figures and images for: Powerful Identification of Cis-regulatory SNPs in Human Primary Monocytes Using Allele-Specific Gene Expression
Source: PLoS One. 2012 Dec 26;7(12):e52260. doi: 10.1371/journal.pone.0052260 (PMC3530574; doi:10.1371/journal.pone.0052260)

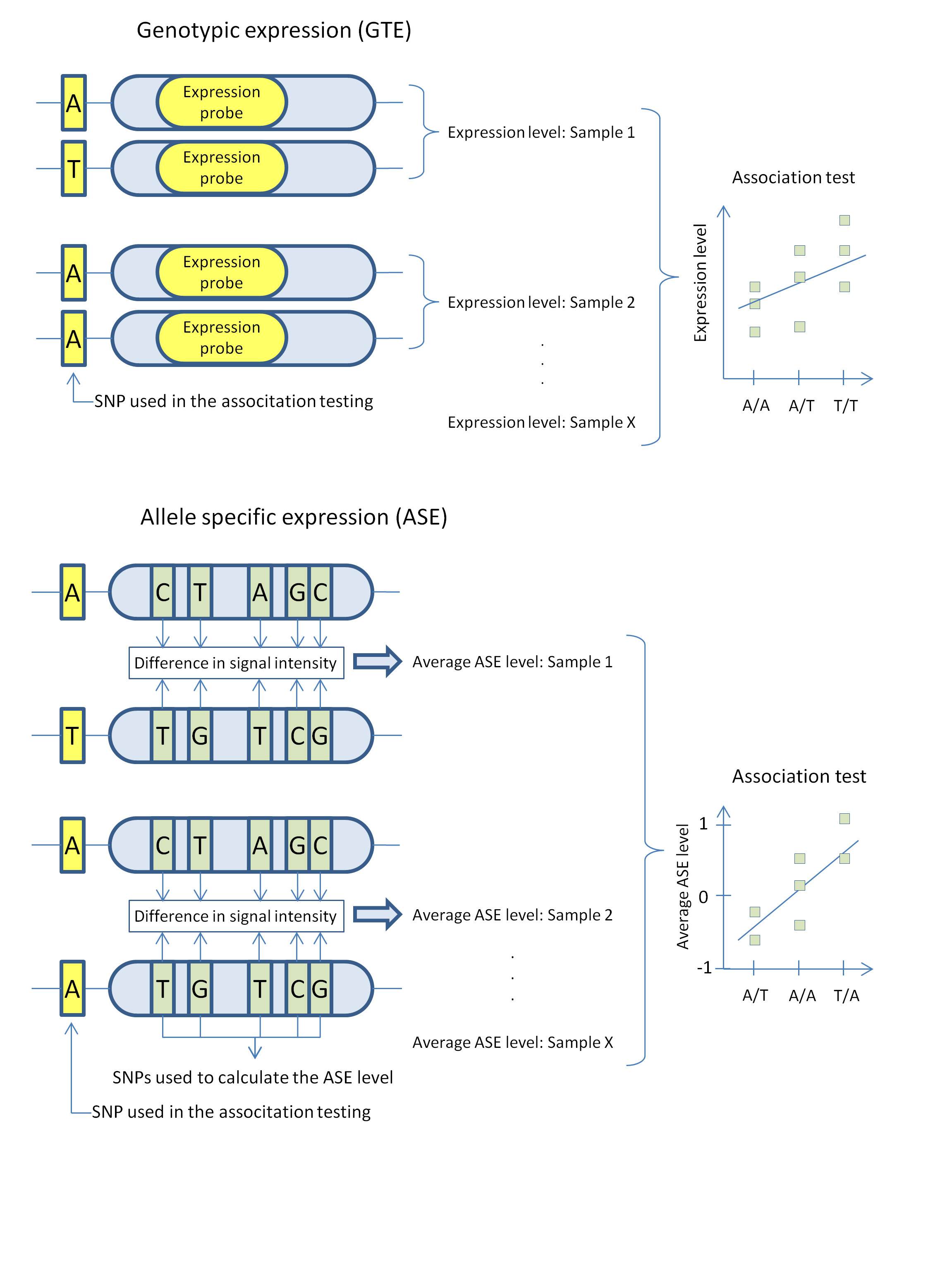

Supplement: Figure S1 — Schematic picture of the principles of GTE and ASE analysis. In GTE the total expression level is measured by a specifically designed expression probe usually located in a transcript. The effect of different genotypes (shown in yellow) can then be tested against the total expression level of each sample to find regulatory SNPs using a linear regression association test. In ASE the allele-specific expression level is measured by the difference in fluorophore signal intensity between the two alleles in the same sample. The average level is calculated for all heterozygous SNPs in the region. This value is then used when testing whether there is a significant effect of the tested genotype (shown in yellow) in a similar association test as in GTE. (TIF) [file pone.0052260.s001.tif]

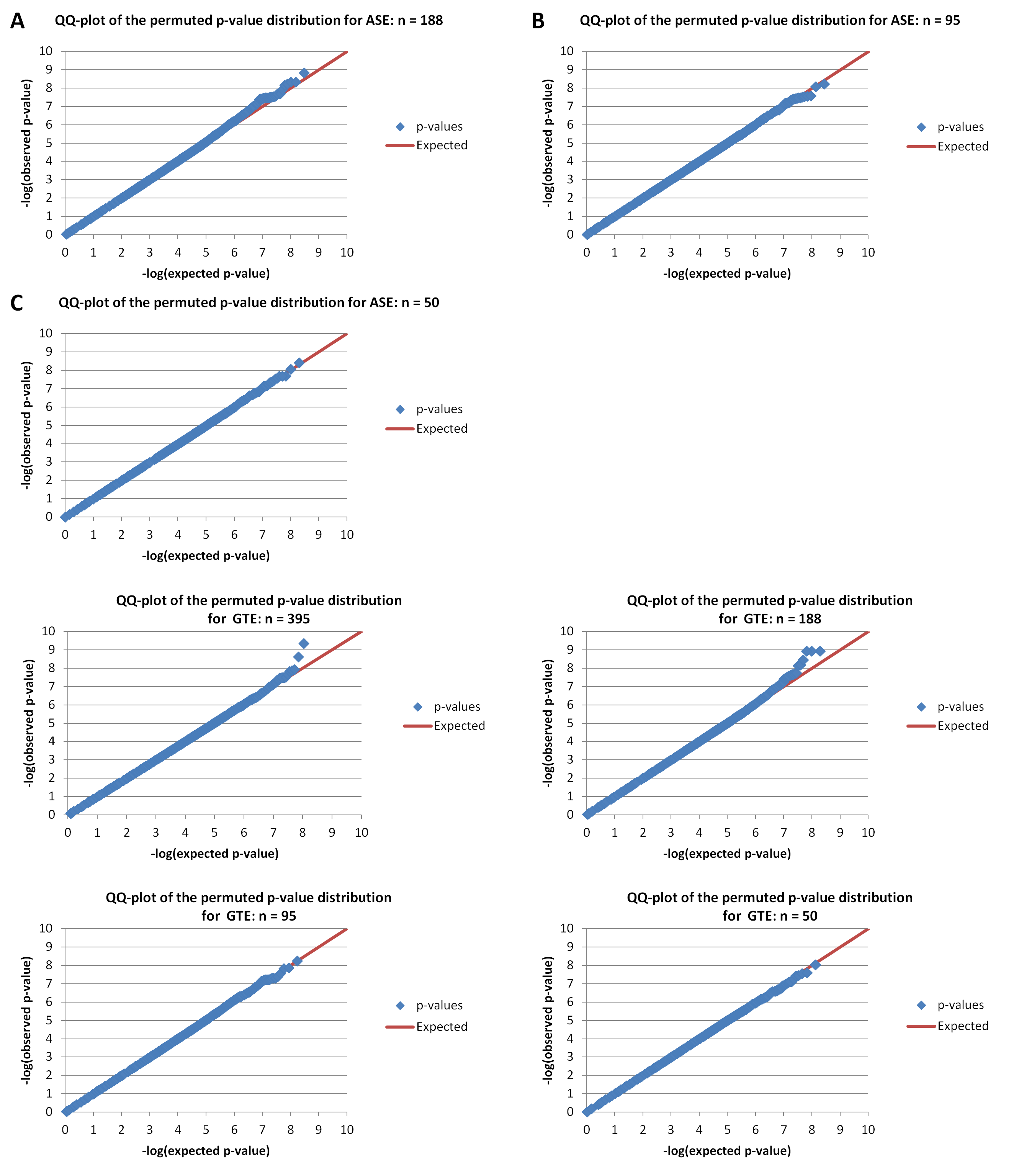

Supplement: Figure S2 — QQ-plots for the permuted p-value distribution compared with a uniform distribution. In Panel A–C, QQ-plots are shown for ASE and in panel D–G QQ-plots are shown for GTE using filtering that removes SNPs with few data points in each genotype group. The permuted data are expected to yield uniformly distributed p-values under the null hypothesis and should therefore follow the red line representing the uniform distribution, which is also the case. However, without filtering, the distribution is starting to skew towards inflated p-values prior to the Bonferroni correction threshold is reached, data not shown. (TIF) [file pone.0052260.s002.tif]

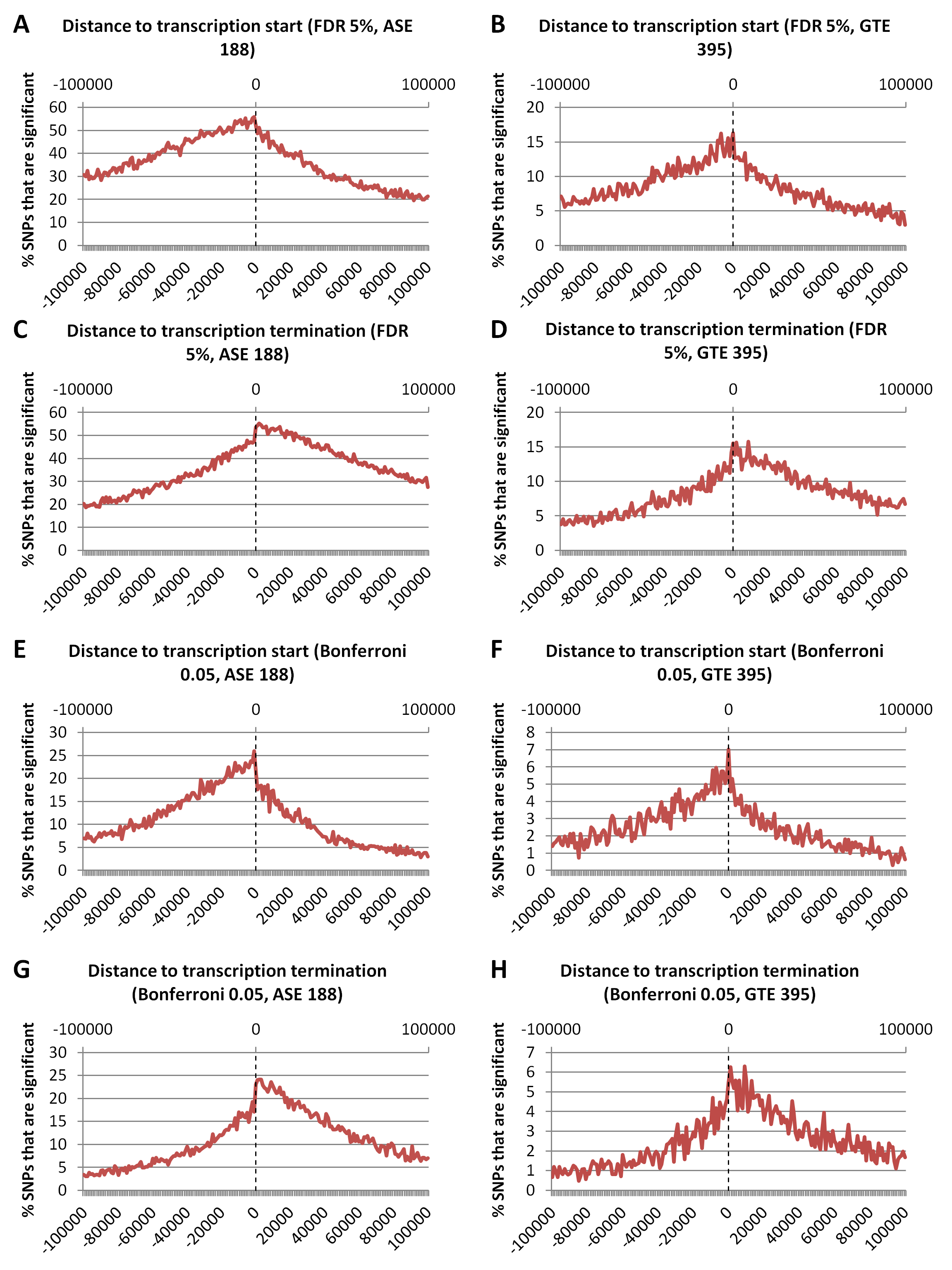

Supplement: Figure S3 — Fraction of rSNPs in relation to the position of TSS and TTS. The fraction of significant association signals compared to the total number of SNPs in 1 kb bins at different distances to the transcription start site (A, B, F, E) and transcription termination site (C, D, G, H) is shown for ASE using 188 samples and for GTE using 395 samples. In A–D the FDR 5% threshold is used and in E–H the Bonferroni 0.05 threshold. All figures show peaks around the start and termination sites. (TIF) [file pone.0052260.s003.tif]
